# Mechanistic PBPK Modelling to Predict the Advantage of the Salt Form of a Drug When Dosed with Acid Reducing Agents

**DOI:** 10.3390/pharmaceutics13081169

**Published:** 2021-07-29

**Authors:** Siri Kalyan Chirumamilla, Venkatesh Teja Banala, Masoud Jamei, David B. Turner

**Affiliations:** Certara UK Limited, Simcyp Division, Level 2-Acero, Sheffield S1 2BJ, UK; venkateshteja.banala@certara.com (V.T.B.); Masoud.Jamei@certara.com (M.J.); David.Turner@certara.com (D.B.T.)

**Keywords:** drug salt, surface pH, acid reducing agents (ARA), PPI, ADAM, absorption, PBPK, Simcyp

## Abstract

Acid reducing agents (ARAs) reduce the dissolution rate of weakly basic drugs in the stomach potentially leading to lower bioavailability. Formulating the API as a rapidly dissolving salt is one strategy employed to reduce the impact of ARAs on dissolution of such drugs. In the present work, a model drug was selected with an immediate release formulation of the free base dosed in both the absence and presence of the ARA famotidine. In the latter case, bioavailability is restricted and several salt formulations were investigated. To simulate these drug products a mechanistic physiologically based pharmacokinetic (PBPK) model was built using the Simcyp Simulator, which illustrates the advantage of formulating an API as a salt compared to the free base form. The simulations use a mechanistic salt model utilising knowledge of the solubility product which was applied to predict the salt advantage. The developed PBPK model exemplifies that it can be critical to account for the surface pH and solubility when modelling the dissolution of low pKa bases and their salts in the gastric environment. In particular, the mechanistic salt model can be used to aid in screening and salt form selection where the aim is to mitigate effects of ARAs.

## 1. Introduction

Poorly soluble weakly basic drugs with low pKa can readily dissolve in the low pH environment typical of the fasted stomach and, provided they do not precipitate in the higher pH conditions of the small intestine, may have sufficient bioavailability (Gesenberg et al., 2019 and references therein). However, under conditions of elevated gastric pH solubility may be limited such that dissolution and therefore relative bioavailability is restricted. Such conditions can arise in the presence of food, in achlorhydric subjects, and where the patient is taking an acid reducing agent (ARA). There is a variety of formulation-based enabling strategies to increase solubilisation of API via different mechanisms including, but not limited to, the use of amorphous solid dispersions, complexing agents, lipid formulations, cocrystals, and using acidifying excipients, as well as formulating the API as a salt. The most appropriate method depends on the physico-chemical properties of the API and a variety of other factors [1,2].

Of these methods, use of acidifying excipients or salt forms lead to supersaturation by changing the pH in the microenvironment of dissolving drug particles. This supersaturation improves the absorption of drug co-dosed with ARAs. For example, Mitra et al. [3] demonstrated formulating with the acidifying agent citric acid along with HCl salt form had similar PK with and without co-dosing of the proton pump inhibitor (PPI) omeprazole in oncology patients, Dickinson et al. [4] used a fumarate salt form to improve PK in achlorhydric patients, Bloomer et al. [5] demonstrated that danirixin exposure was not effected by PPI when formulated as a bromide salt, and Seiler et al. [6] showed that prasugrel hydrochloride has higher exposure when compared to prasugrel free base when dosed with the PPI lansoprazole.

Physiologically based pharmacokinetic (PBPK) modelling has been utilised in the past to predict the pharmacokinetics of salt forms; however, to the authors’ knowledge, a mechanistic salt model coupled to a mechanistic surface pH model has not been applied and published. In a recent publication, Kesisoglou et al. [7] utilised dissolution in biorelevant media to obtain apparent solubility at initial time points (where drug supersaturates) to inform the PBPK model and Gesenberg et al. [8] used in vitro dissolution in SGF and FaSSIF as direct input to a PBPK model for a salt form. The solubility and dissolution advantage of salts is usually linked to favourable changes in the microenvironment pH around the dissolving particles. This pH amongst other factors depends on the buffering capacity of the media driven by type and concentration of buffering species, the concentration of which varies between each segment of the GI tract and changes with prandial state. A mechanistic model to predict the pH on the surface of dissolving salt particles is required to capture the impact of this change in buffer capacity across GI tract and its impact on salt surface pH, solubility and, hence, dissolution rate.

**Figure 1:**
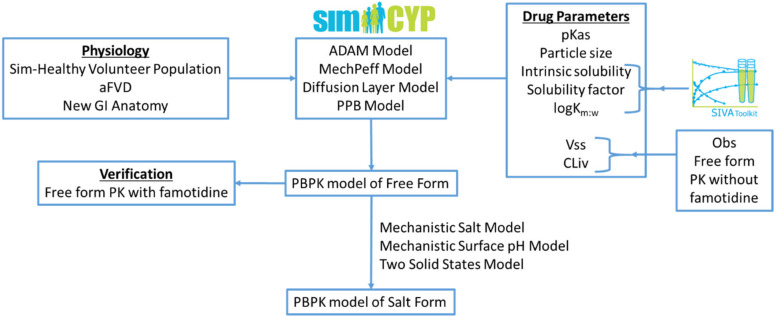
Modelling workflow followed within this research work: aFVD (advanced Fluid Volume Dynamics), PPB (Particle Population Balance), Vss (Volume of distribution at steady state), and CLiv (In Vivo Clearance).

In the present work, a mechanistic salt model, implemented within a PBPK framework, was utilised for the first time to predict the pharmacokinetics of salt forms using equilibrium pH-solubility data; the modelling workflow is illustrated in Figure 1. A model compound selected from the literature, a Bristol Myers Squibb drug candidate (Gesenberg et al. [8]), is a poorly soluble ampholyte with pH-dependent solubility. For the free form of this drug, clinical exposure was found to be acceptable at which point there appeared to be no need to develop a salt form or develop an enabling formulation. However, when the drug was co-dosed with the ARA famotidine there was a significant reduction in exposure. Therefore, Gesenberg et al. conducted a series of studies to “confirm that the observed clinical data were caused by a reduction of dissolution rate and solubility of the drug in a high gastric pH environment and identify an alternate solid form selection approach with a high potential to reduce the pH-effect”. Two salt formulations were investigated in vitro, viz. a hydrochloride (HCl) and a sulphate salt. While both salts were found to dissolve rapidly to give high concentration, the solution created by the HCl salt was found to precipitate after around 20 min. The sulphate salt on the other hand was found to create supersaturated solutions stable for at least three hours in vitro and was therefore chosen to take forward to clinical studies. In a clinical study in dogs, exposure was significantly improved with the sulphate salt compared to the free base when co-dosed with famotidine. Thus, this study provides a rich source of in vitro and clinical data upon which to develop and assess the current PBPK model.

## 2. Materials and Methods

### 2.1. Materials

The Simcyp Simulator (Version 20, Release 1; Certara UK Limited, Sheffield, UK) (the *Simulator*) was used to predict the effect of acid reducing agents on the absorption kinetics of the free and salt forms of a model compound. The Advanced Dissolution, Absorption, and Metabolism (ADAM) model [9,10] with the Minimal PBPK distribution model were used. The PBPK model was developed for the free form of the drug using a middle out approach [11]. Drug physicochemical properties, particle size, and in vitro solubility data were used to predict absorption and clinical pharmacokinetic (PK) data were used to optimise the disposition and elimination of the drug in the fasted state in the absence of acid reducing agents.

The developed model was verified using clinical PK data in the presence of an acid reducing agent, which was not used in the development of the model. The validated model was used for the prospective prediction of the PK of the salt form of the drug in the presence and absence of an acid reducing agent. The Simcyp In Vitro data Analysis toolkit (SIVA Version 4, Release 1; Certara UK Limited, Sheffield, UK) was used for modelling of available in vitro biopharmaceutics experiments. The model drug physicochemical properties, particle size, in vitro solubility data and dog and human clinical PK data were reported by Gesenberg et al. [8].

### 2.2. Modelling of In Vitro Aqueous and Biorelevant Solubility Experiments

Equation (1) shows the solubility equation used in SIVA (and the Simulator) for mechanistic modelling of the aqueous and bile-mediated solubility components of the free form of a drug (the equations for the salt form are given below). In addition, the aqueous solubility of the drug can be limited by its salt solubility, which in this study is captured with solubility factors (*SF*).

(1) $$S_{Tot}=S_{o}\cdot \left(1+\frac{\left[BS\right]}{C_{H_{2}0}}\cdot K_{m:w,unionised}\right)+\cdot S_{i}\cdot \left(1+\frac{\left[BS\right]}{C_{H_{2}0}}\cdot KK_{m:w,ionised}\right)$$

where $S_{Tot}$ is the total solubility (mg/mL) in a given medium; $S_{o}$ is the intrinsic solubility (mg/mL) of the compound (solubility of the unbound, unionised species) in the aqueous phase; $S_{i}$ is the aqueous phase solubility of the ionised species; $\left[BS\right]$ represents the bile salt concentration in a given medium (considered only where the $\left[BS\right]$ > Critical Micelle Concentration (*CMC*)); $C_{H_{2}0}$ is the water concentration (55.56 mM); and $K_{m:w,unionised|ionised}$ are the bile micelle partition coefficients for neutral and ionised drug species, respectively.

Aqueous solubility at pH 7.5 is used to back-calculate the intrinsic solubility of the drug-pKa1 was fitted to explain the aqueous solubility from pH 7.9 to 3.9 using the reported pKa1 as an initial estimate. The salt *SF* was estimated from the solubility at pH 1.5. Note that the *SF* is used to define the maximum aqueous phase solubility viz. *max*(*S_o_* + *S_i_*) = *SF* · *S_o_*.

The partition coefficients of the drug between water and bile salt micelles for the neutral species (*K_m:w, unionised_*) and ionised species (*K_m:w, ionised_*) are estimated from the solubility of the drug in biorelevant media (FaSSIF and FeSSIF media) using the partition coefficients predicted from logP_o:w_ for the initial estimates. Table 1 lists the aqueous and biorelevant solubility values of the model drug.

### 2.3. PBPK Model Development for the Free Form of the Drug

The PBPK model for the free form of the drug was developed using the Simcyp Human Simulator V20. Drug physicochemical properties, particle size, and in vitro solubility data were used to predict absorption and clinical PK data were used to estimate the disposition and elimination parameters of the drug.

The Immediate release (IR) solid formulation option with the Diffusion Layer Model (DLM) was used to describe the dissolution of API particles. The diffusion layer model (Equation (2)) is based on an extension of the Wang and Flanagan diffusion layer model [12].

(2) $$DR\left(t\right)=\overset{2}{\underset{SS=1}{\sum }}\overset{NBINs}{\underset{i=1}{\sum }}N_{i,SS}S_{DR,SS}\frac{D_{eff,SS}\left(t\right)}{h_{eff,i,SS}\left(t\right)}4\pi a_{i}\left(t\right)\left(\left(a_{i}\left(t\right)+h_{eff,i,SS}(t\right)\right)\left(\left(S_{surface,SS}\left(t\right)-C_{bulk,\;ss}(t\right)\right)$$

where $DR\left(t\right)$ is the overall dissolution rate at time *t*; *SS* refers to different solid states (e.g., crystal polymorphs or, as in this case study, the free and salt forms); $N_{i,SS}$ is the number of particles (in the *i*th particle size bin for a polydispersed formulation with *NBIN_S_* particle size bins); $S_{DR,SS}$ is an empirical scalar (default value 1) for each of the dissolving solid states used to either increase or decrease the dissolution rate based on the observed in vitro dissolution rate (also referred to as the DLM scalar); $D_{eff,SS}$*(t)* is the effective diffusion coefficient in the diffusion layer at time *t* for each of the solid states; $a\left(t\right)$ is particle radius at time *t*; $h_{eff,SS}$*(t)* is the effective diffusion layer thickness at time *t* for each of the solid states; $C_{bulk,SS}\left(t\right)$ is the concentration of the drug in bulk solution at time *t* for each of the solid states*;* and $S_{surface,SS}\left(t\right)$ is the solubility of drug at the particle surface at time *t* for each of the solid states. Within the Simulator, Equation (2) is used within the relevant ordinary differential equation with respect to particle radius changes over time.

A Particle Population Balance (PPB) model is used to handle fine particle transit (dispersion along the GI tract), dissolution, and precipitation. The precipitation of dissolved drug was handled using the Simulator precipitation Method 2 [10] and default values are used for critical supersaturation ratio (*CSR*) and precipitation rate constant (*PRC*) parameters as these values were able to capture overall plasma concentration profiles of the free form in the absence of famotidine co-dosing (see Results section).

The Simulator Mechanistic Permeability (MechPeff) model [13] was used to predict the regional effective gut wall permeability (*P_eff,man_*) of the model compound for which the drug intrinsic (neutral species) membrane permeability (*Ptrans*,*0*) is a key parameter. The model considers drug parameters (including the aforementioned *Ptrans*,*0*, ionization, diffusion coefficient, micelle partitioning, and molecular radius) and gut physiology (regional villus morphology, *plicae circulares*, unstirred boundary layer (UBL) thickness, UBL local pH and paracellular pore radius). The model incorporates interindividual variability of regional *P_eff,man_*, the evidence for the existence of which is provided by loc-I-gut model experiments, for example Lindahl et al. [14]. *Ptrans*,*0* was predicted using the model compound logP_o:w_ value with the in-built correlation function in the Simulator v20. Under conditions of time variant pH, bile salt concentrations and thus solubility regional *P_eff,man_* is recalculated at every time point during the simulation.

The SIM-Healthy Volunteer population is used with default settings except for the selection of the advanced Fluid Volume Dynamics (aFVD) model and the “New Anatomy” option for the small intestine. The aFVD model is based on an updated meta-analysis of published GI lumen baseline fluid volumes. Compared to the older Fluid Volume Dynamics (FVD) model of the Simulator, among other things, the aFVD model accounts for the fluid reabsorption in duodenum and has lower average subject baseline fluid volumes (31.36, 85.8, and 11.92 mL in the stomach, small intestine, and colon respectively—the corresponding values for the FVD model are 53.32, 139.35, and 13 mL). Both FVD models are dynamic in the sense that when a drink of water is taken (with or without drug) the volume is added into the stomach, which is then dynamically propagated into and along the small intestine and progressively absorbed until baseline volumes are regained. As with most physiological parameters available in the Simulator, there is interindividual variability of these baseline volumes and of the rate of absorption of ingested water [9].

The “New Anatomy” option for the small intestine was implemented in the Simulator Version 20 where the diameter and length of small intestine and colon were changed based on a recent extensive meta-analysis of literature data using in situ data only. In addition, and consequent to the diameter/length changes, the *plicae circulares* fold expansion, regional percentage of villous blood flow and small intestinal regional transit times were updated. The main impact of the diameter and *plicae circulares* changes is that predicted absorption rate is significantly increased which results in improved prediction of fa for many APIs—further details of these updates are to be described in a separate publication (ms in preparation) while all parameter values are available in the Simulator databases.

The fraction unbound in plasma (fu) and blood to plasma ratio (BP) are predicted using the Simulator inbuilt QSARs; these values were not reported by Gesenberg et al. The Minimal PBPK model was used to describe the tissue distribution of the drug and in vivo IV clearance was used to describe the elimination of the drug. The volume of distribution at steady state (V_ss_) and in vivo clearance (CLiv) were optimised from the observed oral clinical PK data of the model compound in the absence of the acid reducing agent. As the drug is primarily metabolised by the CYP3A4 enzyme [8], the “percentage CL_H_ by 3A (%)” was set to 100 to enable capture of gut first pass metabolism. The fraction unbound in the enterocytes (fu_gut_) was assigned the default value of 1, which assumes no binding in enterocytes as it represents the worst case scenario for gut first pass metabolism (however, simulated Fg was very close to one so the value of fu_gut_ is not critical to the outcomes).

The developed PBPK model was used to simulate 10 trials of 10 individuals (i.e., 100 different virtual subjects) using the SIM-Healthy Volunteer population dosed with 150 mg of the model drug under fasting conditions without ARA co-dosing; i.e., with default gut pH values. Table 2 lists the parameters used for building the mechanistic absorption and disposition model for the drug free form.

### 2.4. Drug Free Form PBPK Model Verification: Simulations with the Acid Reducing Agent—Famotidine

The performance of the PBPK model developed for the free base form of the drug to predict the clinically observed famotidine effect was verified by simulating 10 trials of 10 individuals using the built-in SIM-Healthy Volunteer population. The Stomach, Duodenum, and Jejunum pH values were manually changed to 7.2 based on observed pH measurements in the stomach and upper small intestine when famotidine was dosed two hours prior to measurement [15]; note these pH measurements were made in a different set of subjects to those used in the model drug PK studies. The between subject variability expressed as a percentage CV was set to 2, 5, and 5% for the stomach, duodenum, and jejunum respectively so as to closely match the observed pH value ranges in the famotidine treated subjects. The median (range) pH values generated by the Simulator using the above mean and CV are 7.2 (6.7–7.5), 7.2 (6.5–8.1), and 7.2 (6.3–8.1) for the Stomach, Duodenum, and Jejunum 1 gut segments, respectively. These values are close to the observed median (range) pH values of 7.2 (6.9–7.3) and 7.2 (6.6–8.3) measured ten minutes after 240-mL water administration to the stomach and thirty minutes after 240-mL water administration to the lower small intestine, respectively [15].

The predicted PK parameters C_max_, AUC_0–24,_ and T_max_ were compared against the observed plasma PK parameters when 40 mg of Famotidine was dosed two hours prior to dosing of 150 mg of the free form of the drug [8].

### 2.5. PBPK Model Development and Simulations of the Salt Formulation

The mechanistic salt model implemented in the Simulator V19 was used to model salt solubility and dissolution. This model includes consideration of the salt solubility product to handle solubilisation of the salt coupled to a mechanistic surface pH model to handle the dissolution rate advantage; further details are given below. Table 3 shows the additional input parameters for the salt model. The highest observed aqueous solubility of the free form is used as the salt solubility at *pH_max_* based on the assumption that the solubility at lower pH values is due to salt formation during the solubility experiments. A sensitivity analysis was performed using a range of salt solubility at *pH_max_* from 0.65 to 65 mg/mL (i.e., 10-fold above and below the solubility at the selected *pH_max_*). The simulated plasma concentration profiles, both with and without famotidine co-dosing, were not sensitive within this range. The Simulator back calculates the *K_sp_* from the salt solubility at *pH_max_* and uses it for the calculation of salt solubility below *pH_max_* for a basic drug and above *pH_max_* for an acidic drug. For an ampholytic drug, the calculation depends on the type of counterion. Above *pH_max_* for a base and below *pH_max_* for an acidic drug, the pH-solubility is calculated using the free form intrinsic (i.e., unbound, unionised) solubility as the model assumes instantaneous conversion of the salt to the free form (above *pH_max_* for a base and below *pH_max_* for an acid) as is frequently observed during particle dissolution experiments [16,17].

The equation used by the Simulator to calculate salt solubility depends on the type of drug (acid/base/ampholyte), type of counterion (acid/base), *pKa*(s) of the counterion (weak/strong), and drug-counterion stoichiometry (1:1/1:2 salt). Equations (3)–(5) show the salt solubility calculations for a 1:1 salt of a diprotic basic drug with a strong acid counterion.

(3) $$S_{surface}\left(t\right)=S_{o}\cdot S_{o_{scalar}}\left(t\right)\cdot \left(1+\frac{\left[BS\right]\left(t\right)}{C_{H_{2}0}}\cdot K_{m:w,unionised}\right)\;+\cdot S_{i}\left(t\right)\cdot \left(1+\frac{\left[BS\right]\left(t\right)}{C_{H_{2}0}}\cdot K_{m:w,ionised}\right)\;\mathrm{Below}\;{pH}_{max}$$

(4) $$S_{i}\left(t\right)=\frac{K_{sp}}{\sqrt{Ksp}+\left[Endogenous\;counterion\right]}\left(1+10^{pKa2-pHsurface\left(t\right)}\right)\;\mathrm{Above}\;{pH}_{max}$$

(5) $$S_{i}\left(t\right)=S_{0}\cdot \left(10^{pKa1-pHsurface\left(t\right)}+10^{pKa2-pHsurface\left(t\right)}+10^{pKa1+pKa2-2pHsurface\left(t\right)}\right)$$

where *pKa*2 is the lowest value *pKa* of the diprotic base and $\left[Endogenous\;counterion\right]$ is the concentration of endogenous counterion in the GI tract fluids. The Simulator model includes dynamic concentrations of the endogenous counterions $Cl^{-},\;Na^{+},\;K^{+},\;\mathrm{and}\;Ca^{2+}\;$ that can act as common ions where, for example, a drug formulated as a chloride salt is dosed. Endogenous ion concentrations are diluted when a drink of water is taken, gradually returning to baseline levels over time.

A mechanistic surface pH model is used for the calculation of surface solubility (*S_surface_*) of the dissolving salt. This model is an extension of the pseudo-steady state approach of Ozturk et al. [18,19] for predicting surface pH (*pH_surface_*) adapted to deal with pharmaceutical salts. The model recalculates *pH_surface_* (and therefore *S_surface_*) at very small time intervals (default 36 s, but adjustable if required) during the simulation. An iterative Newton Solver is used to solve the required equations. The model is sensitive to buffer capacity expressed via in vivo regional bicarbonate concentrations with population variability (and much higher values under fed conditions) derived from literature meta-analysis.

**Figure 2:**
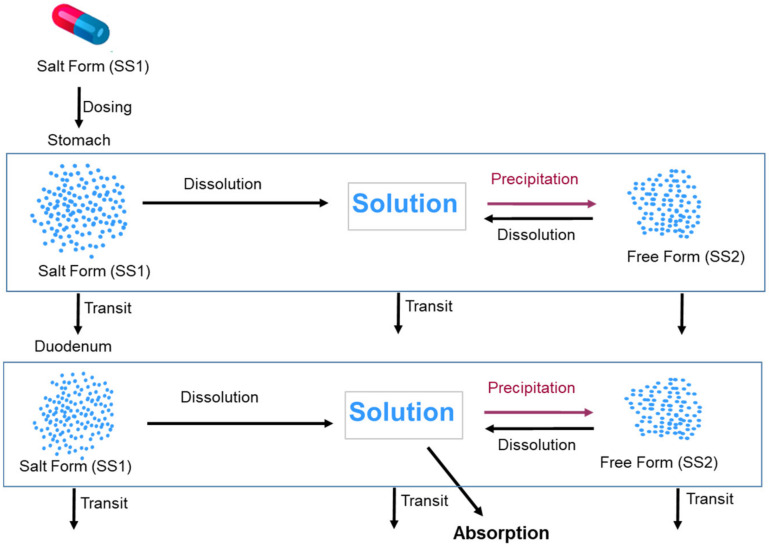
Modelling approach taken to predict the absorption of the salt form of the model compound utilizing the two Solid States model in the Simulator. The figure shows only stomach and duodenum compartments, similar processes occur in other segments of the GI tract.

The two Solid States model is used to handle supersaturation and precipitation (if suitable conditions arise) to the free form of the drug once the salt form is dissolved. For this, Solid State 1 (SS1) is set to “Salt Form” and Solid State 2 (SS2) is set to “Free Form” (in this instance the free base) within the Simulator. Since the drug is dosed in the salt form, initially the total dose is assigned to the Solid State 2 (SS2) and no free base. During the course of the simulation, these are dynamically changed due to dissolution and precipitation events. This facility can be used to define intermediate proportions of salt and free base where, for example, disproportionation has occurred during storage. It can also be used to define relative proportions of two polymorphs or a polymorph and amorphous drug. The modelling approach taken to predict absorption from the salt form of the model compound is illustrated in Figure 2.

The “Drug:Counterion Stoichiometry” is set to 1:1 and sulphate is selected as the counterion. The Simulator has a database of a range of commonly used counterions in salt formulations including the Type and pKa values for sulphate listed in Table 3.

Precipitation to SS1 was blocked while precipitation to SS2 was permitted, with the assumption that dissolved drug does not precipitate back to the salt form and precipitation, should it be predicted to occur (see Results section), is to the free form—see Discussion section for further explanation. Precipitation to the free base form was handled using the Simulator precipitation Method 2—default values are used for the Critical Supersaturation Ratio (*CSR*) and Precipitation Rate Constant (*PRC*) parameters and the same values were used in the PBPK model where the drug was dosed in the free form.

The developed PBPK model of salt form was used to predict plasma concentrations by simulating 10 trials of 10 individuals using the built-in SIM-Healthy Volunteer population dosed with 150 mg of salt form of the model drug under fasting conditions without (using default gut pH values) and with (using gut pH values mentioned in Section 2.4) ARA co-dosing.

## 3. Results

### 3.1. Modelling of In Vitro Aqueous and Biorelevant Solubility Experiments

The model compound is an ampholyte with two basic pKas (6.5 and 2.1) and an acidic pKa (9.8). However, the acidic pKa is significantly above gastrointestinal physiological gut pH range for a typical subject (range 1.5–7.4) and even for extreme subjects within a large population pH rarely exceeds 8 [20]. Thus, the ionization due to the acidic group is negligible for most subjects while the two basic pKas are within the physiological pH range and thus relevant to ionization calculations. Therefore, the model compound is treated as a diprotic base for modelling purposes. Thus, the in vitro aqueous solubility values measured at higher pH (pH 8.5, pH 9.1, and pH 10.6) are not used in the solubility modelling exercise (as the solubility values at these pH values are expected to be influenced by the high acidic pKa).

**Figure 3:**
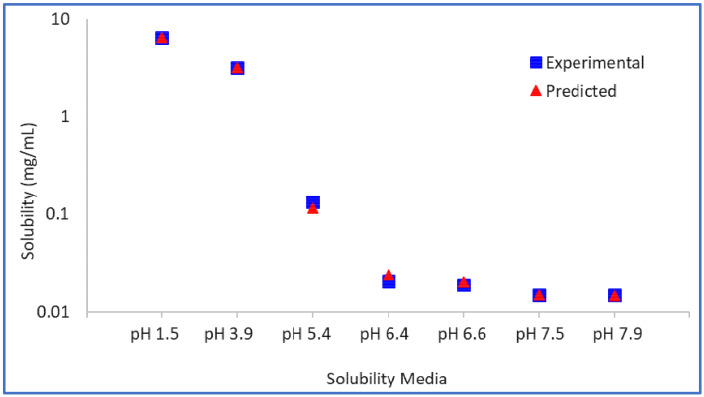
Model drug free form experimental vs. predicted aqueous solubility.

The intrinsic solubility (*S_o_*) of the model compound is found to be 0.0142 mg/mL from back calculation, and the value of pKa1 is estimated to be 6.25, which is close to the measured pKa1 of 6.5. The *SF* was estimated to be 457.8. Using these parameters, the predicted aqueous solubility matches the experimental solubility very closely (Figure 3).

**Figure 4:**
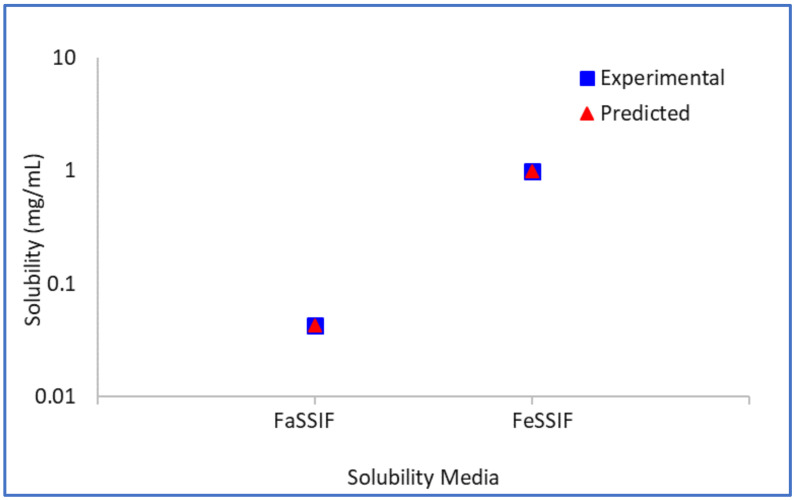
Model drug free form experimental vs. predicted biorelevant solubility.

The logarithm (base 10) of the bile–micelle partition coefficients for unionised and ionised species is estimated to be 4.34 and 3.97, respectively. Using these partition coefficients (and the previously estimated or confirmed *S_o_* and pKa values) the predicted solubility in FaSSIF and FeSSIF media match closely the experimental solubility values in these media (Figure 4). No other data were available to verify further these parameters.

### 3.2. Simulation and Prediction of Systemic Plasma Concentration Profiles of Free Form of Drug

**Figure 5:**
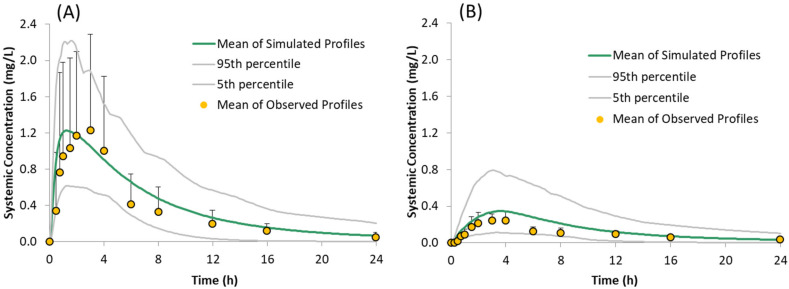
Simulated (Mean, 5th and 95th Percentiles) and Observed (Mean + SD) plasma concentration time profiles of the model compound following oral administration of 150 mg of the free form under fasted conditions: (**A**) without famotidine and (**B**) with famotidine, as reported by Gesenberg et al. [8].

The performance of the PBPK model in humans with the free form was assessed by simulating the systemic plasma concentration profiles of the model compound under fasting conditions in the absence of acid reducing agents, i.e., using default gut pH values. Overlays of the observed and simulated PK profiles of the model compound are shown in Figure 5A. The results demonstrate that the simulated PK profiles adequately reproduced the observed data. The predicted/observed ratios for the mean profile are within 1.5 fold for PK parameters C_max_ and AUC_0–t_ (Table 4).

The developed model was verified by predicting the plasma concentrations in the presence of the ARA famotidine. The model was able to explain the observed lower concentrations adequately (Figure 5B). The predicted/observed ratios are within 1.5 fold for PK parameters C_max_ and AUC_0–t_ (Table 3).

### 3.3. Prediction of Systemic Plasma Concentration Profiles of Salt Form of Drug

**Figure 6:**
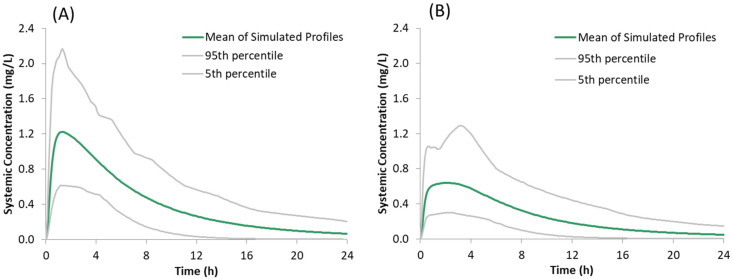
Simulated plasma concentration time profiles of the model compound following oral administration of 150 mg of the salt form under fasted conditions: (**A**) without famotidine and (**B**) with famotidine co-dosing.

**Figure 7:**
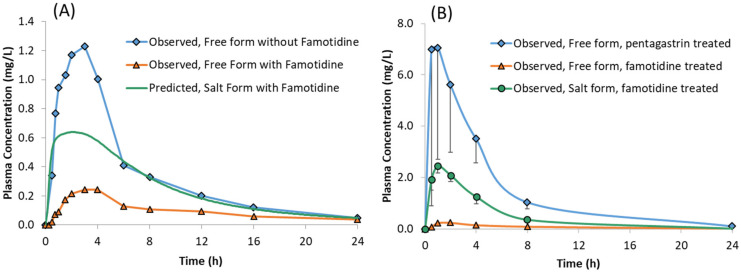
Plasma concentrations of model compound free form with and without famotidine and the salt form with famotidine in: (**A**) Healthy Human volunteers and (**B**) Male Beagle Dogs.

The predicted plasma concentrations for the model compound dosed as a sulphate salt with and without famotidine co-dosing are shown in Figure 6 and the corresponding PK parameters are given in Table 5. The predicted plasma concentrations in the presence of famotidine are in line with the dog PK where the plasma concentration of the salt form dosed in famotidine treated dogs is higher than those obtained for the free form dosed in famotidine treated dogs but lower than those obtained for the free form dosed in pentagastrin treated (absence of ARA) dogs (Figure 7B), verifying the predictability of the developed model with the assumption that the trend observed in dogs also applies to humans.

## 4. Discussion

### 4.1. Prediction of the Effect of ARAs on the Absorption of the Free Form of the Model Compound

Acid reducing agents when co-dosed can significantly reduce dissolution and absorption of weakly basic drugs, thereby leading to sub therapeutic plasma concentrations. In the present work, the PBPK model developed for the free form of the drug was verified by predicting the effect of ARAs on the pharmacokinetics of a model compound. The model compound is a poorly soluble ampholyte with pH-dependent solubility. In the ARA study when 40 mg of famotidine, an ARA which acts by blocking histamine H_2_ receptors in the stomach, is dosed 2 h prior to dosing of 150 mg the model compound, the plasma concentration of the model compound reduced significantly. When compared to plasma concentrations without famotidine dosing, the C_max_ was reduced by four-fold and AUC_0–24_ was reduced by two-fold, approximately [8].

Although acid reducing agents such as PPIs and histamine H2 receptor antagonists reduce acid production, buffer capacity, chloride ion concentration, osmolarity, and surface tension in stomach and increase pH in upper small intestine [15], only changes to the stomach, duodenum and jejunum pH were used in the model.

The developed model was able to predict the reduced plasma concentrations resulting from reduced solubility and dissolution rate in the stomach.

**Figure 8:**
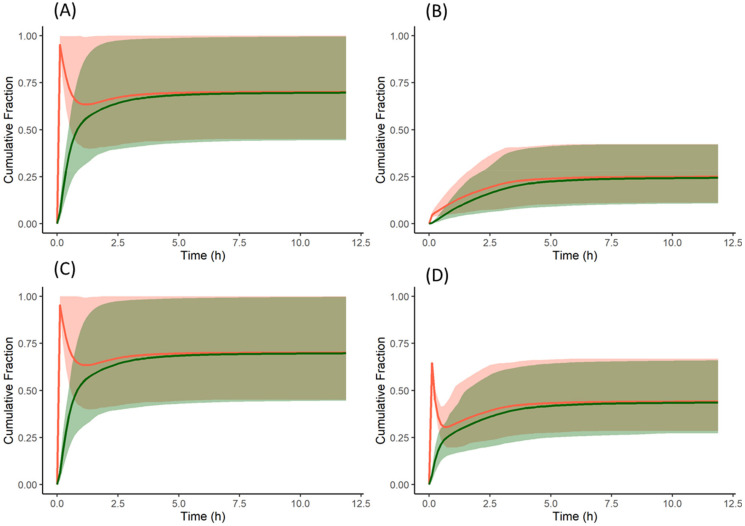
Cumulative fraction of dose dissolved (red) and cumulative fraction of dose absorbed (green) profiles of the model compound: (**A**) free form without famotidine, (**B**) free form with famotidine, (**C**) salt form without famotidine, and (**D**) salt form with famotidine. Solid lines represent the mean and shaded area represent 5th and 95th percentiles of individual profiles. A reduction in the % dissolved reflects precipitation. For dispersible formulations as in this example the % dissolved reflects the net effect across all nine segments of the ADAM model.

The free form of the drug, when dosed without famotidine, dissolves in the stomach due to the acidic pH and then precipitates once the drug enters the duodenum—as reflected the cumulative fraction dissolved profile (Figure 8A). However, while the drug continues to dissolve in the stomach, the dissolved concentration available for absorption in the duodenum remains high until the concentration reduces due to precipitation. This leads to higher absorption when compared to subjects pre-treated with famotidine. In subjects pre-treated with famotidine, there is an increase in stomach pH and solubility and dissolution rate in the stomach are reduced (Figure 8B) resulting in lower concentrations of drug available for absorption in the duodenum. The solubility of the model compound in the stomach (of an average subject) without and with famotidine co-dosing is 6.5 and 0.0166 mg/mL, respectively, which for the latter case results in a slower dissolution rate and lower plasma concentrations.

While studying the effect of ARAs on the pharmacokinetics of drugs, it is also important to consider metabolic- and transporter-mediated drug–drug interactions (DDIs). As the model compound is primarily metabolised by CYP3A4 [8] and famotidine has minimal potential for CYP450-mediated DDIs [21,22], metabolic DDI was not considered in this study. Famotidine is also an inhibitor of organic cation uptake transporters (OCT) [23]. However, there is no evidence that the model compound is a substrate of OCT transporters and therefore transporter mediated DDIs are assumed absent or insignificant in this study.

### 4.2. Prediction of the Effect of ARAs on the Absorption of the Salt Form of the Model Compound

**Figure 9:**
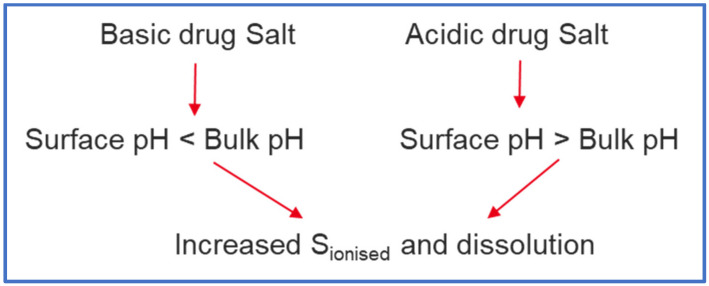
Effect of Surface pH on drugs formulated as salts (‘Salt Form’).

While there are several methods employed to reduce the effect of ARAs on dissolution of basic drugs [2], development of a salt formulation is one of the strategies frequently employed. A salt, fully ionised at the point of dissolution, can dissolve rapidly and to a greater extent (depending on dose) than a partially ionised low pKa base under elevated pH conditions such as those found in presence of food, achlorhydric subjects, or ARAs. While surface pH effects under conditions of insufficient buffer capacity—typical of the fasted stomach—can restrict the dissolution of free acids or bases, for pharmaceutical salts the opposite is often true; i.e., surface pH effects can promote rather than limit salt dissolution (Figure 9). The combined effect of these two factors (full ionisation and surface pH effects) means that salt dissolution can drive the creation of supersaturated solutions with respect to the equilibrium solubility of the free base and the salt form.

In the present work, a PBPK model developed and verified for the free form of the model compound was extended to predict the plasma concentrations of the model compound dosed as a sulphate salt in the presence and absence of famotidine co-dosing using the Simulator mechanistic salt model.

In the absence of famotidine co-dosing, the PBPK model for the salt form of the model compound predicted similar plasma concentrations to those observed for the free form. The cumulative dissolution profile of the salt form is similar to that of the free form. As with the free form, when the salt is dosed, the entire dose (150 mg) is dissolved in the stomach, which upon entering the duodenum forms supersaturated solutions and precipitates as the free form (Solid State 2). As the precipitate is not the salt form, the precipitated drug does not benefit from the favourable salt surface pH to re-dissolve. Therefore, the dissolution of the salt form is similar to the free form in the absence of famotidine co-dosing.

For the salt form PBPK model, precipitation to the salt form from supersaturated drug solution was blocked, as the precipitation to the salt form would require the product of molar concentrations of counterion and free drug to be higher than the *K_sp_* of the salt. A priori, this is unlikely due to the dilution of the sulphate counterion in the GI tract fluids and fluid taken with the dose (240 mL). In order to test this assumption, simulations were performed without blocking precipitation to the salt form and the plasma concentrations were found to be very close to plasma concentrations where precipitation to the salt form was blocked. 

When famotidine is co-dosed, the PBPK model for the salt form of the model compound predicted higher plasma concentrations than observed for the free form co-dosed with famotidine. However, the plasma concentrations are lower than observed for the free form without famotidine co-dosing. The model predictions for humans are consistent with those observed in preclinical dog PK studies where the plasma concentrations for the salt form dosed in famotidine treated dogs are higher than those obtained for the free form dosed in famotidine treated dogs but lower than those obtained for free form dosed in pentagastrin treated dogs (with low gastric pH) (Figure 7B). In the absence of human clinical data for the salt formulation, these observations can be taken to verify the predictability of the developed model in humans subject to the assumption that the trend observed in dog studies applies to humans.

**Figure 10:**
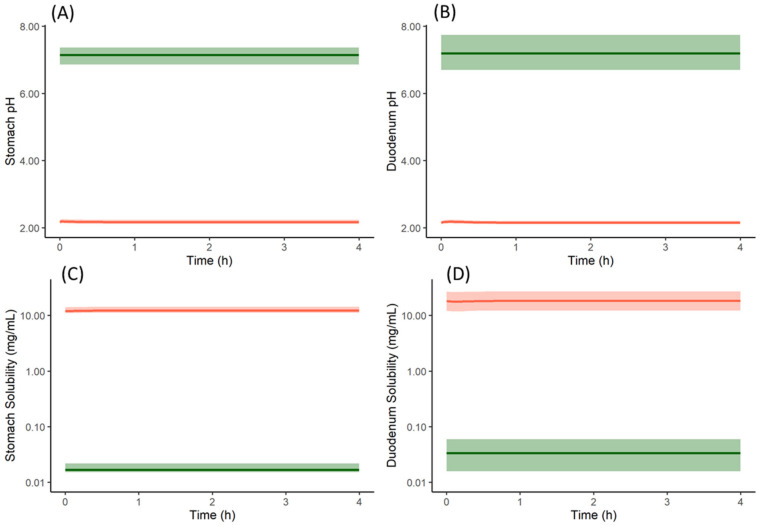
Particle surface pH (red in (**A**,**B**)), bulk fluid pH (green in (**A**,**B**)), particle surface solubility (red in (**C**,**D**)) and bulk fluid solubility (green in (**C**,**D**)) profiles of model compound salt form (Solid State 1) with famotidine in stomach (**A**,**C**) and duodenum (**B**,**D**). Solid lines represent mean and shaded area represent 5th and 95th percentiles of 100 simulated individual profiles. The profiles are only shown for stomach and duodenum compartments, similar results are obtained for the other intestinal segments.

**Figure 11:**
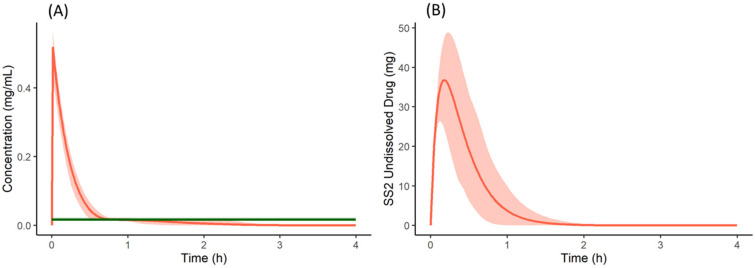
(**A**) Total concentration (Red) and bulk fluid equilibrium solubility (green) of the drug in stomach when co-dosed as a salt form with famotidine. (**B**) Undissolved (precipitated) drug of Solid State 2 (free form) in the stomach when the salt form of the drug is co-dosed with famotidine. Solid lines represent mean and shaded area represent 5th and 95th percentiles of 100 simulated individual profiles.

From the cumulative dissolution profiles generated by the Simulator, it can be seen that the dissolution is higher when the drug is dosed as a salt with famotidine (Figure 8D) compared to drug dosed as the free form with famotidine (Figure 8B). This is due to lower pH on the surface of the salt form drug particle (red in Figure 10A,B) when compared to bulk fluid (green in Figure 10A,B). This lower surface pH leads to higher solubility on the particle surface (red in Figure 10C,D) compared to bulk fluid solubility (green in Figure 10C,D). This higher solubility at the dissolving surface leads to higher dissolution rate of the salt form dosed with famotidine. However, as particle dissolution is driven by difference between surface solubility and bulk fluid concentration (Equation (2)), the bulk fluid concentration exceeds the bulk equilibrium solubility of the drug (as bulk solubility of drug is lower than surface solubility). This leads to supersaturation and ultimately precipitation of the drug in the stomach. Figure 11A shows the total concentration of drug exceeding the bulk solubility in the stomach when dosed as salt in the presence of famotidine and Figure 11B shows the formation of Solid State 2 (free form) in stomach due to precipitation and its subsequent transit.

**Figure 12:**
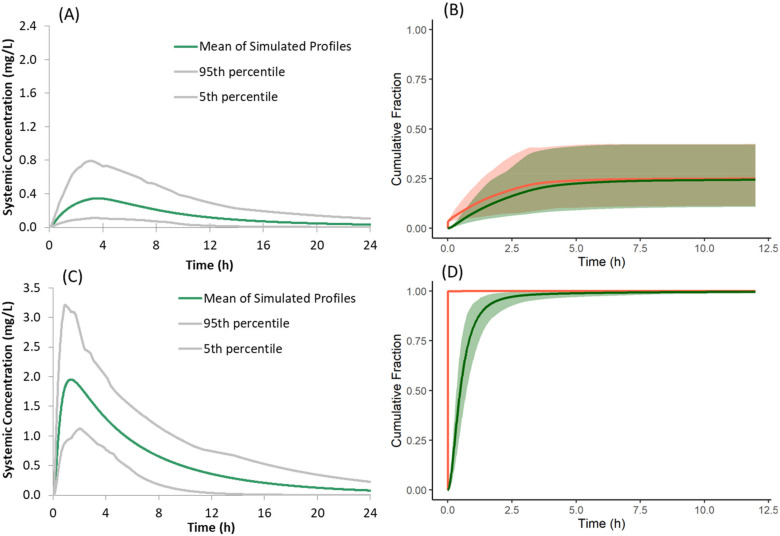
(**A**,**C**) Simulated plasma concentration time profiles and (**B**,**D**) cumulative fraction of dose dissolved (red) and absorbed (green) of the model compound following oral administration of 150 mg of the salt form under fasted conditions with famotidine co-dosing when the mechanistic Surface pH model is not selected (**A**,**B**); when Solid State 2 (free form) is not used in the model (**C**,**D**).

When the mechanistic surface pH model was not used in the PBPK model of the salt form co-dosed with famotidine, the dissolution rate, absorption rate, and plasma concentration profiles of the salt form (Figure 12A) were similar to that of the free form with famotidine. This deviates from the observed PK of the salt form in preclinical dog studies where the plasma concentrations of the salt form dosed with famotidine are higher than the free form dosed with famotidine. At least within our model, this illustrates the importance of incorporating the mechanistic surface pH model to simulate/study the dissolution advantage of a salt form over the free form when subjects are dosed with ARAs.

While the present model in the Simulator can predict the surface pH of dissolving salt particles based on the drug and counterion properties and buffer capacity, the microenvironment pH of dissolving salt can also be influenced (if present) by acidic (e.g., citric acid) and basic (e.g., meglumine) excipients in the formulation. If these excipients are used, it is recommended to use the measured microenvironment pH of the formulation and use it as direct input via the “User-defined Surface pH” option of the Simulator rather than using the mechanistic model.

In the present model for the salt form, both the salt and free forms are simultaneously simulated to capture the precipitation of dissolved drug from supersaturated solution to the free form. When the free form is not selected as SS2, and supersaturated drug solution is allowed to precipitate as the salt form, the predicted plasma concentrations of the salt form dosed with famotidine are greater than those observed with the free form, both with and without famotidine co-dosing (Figure 12C). This deviates from the observed PK of the salt form in preclinical dog studies where the plasma concentration of salt form dosed with famotidine is lower than that of the free form dosed without famotidine. These higher simulated plasma concentrations are the result of instantaneous redissolution of the precipitated salt due to its elevated surface solubility (due to lower surface pH) of the salt form in all segments of the GI tract (Figure 12D). This observation points to the importance of simulating both salt and free forms simultaneously to allow precipitation of dissolved drug from supersaturated solutions to the free form when modelling absorption kinetics of the salt form.

## 5. Conclusions

A mechanistic human PBPK model has been built for a model low pKa basic drug dosed as both a free base and a sulphate salt in the presence and absence of the acid reducing agent famotidine. In the presence of the latter, gastric pH is significantly elevated and for the free base bioavailability is significantly reduced. In canine studies, a sulphate salt of the drug provided significant recovery of bioavailability. The developed PBPK model was able to recover the human PK studies for the model drug with and without co-dosed famotidine for the free base. For the sulphate salt, this verified model predicted significant enhancement of bioavailability in humans that qualitatively matched the enhancement observed in dog studies. Critical to these predictions was the application of a mechanistic salt and surface pH model to drive creation of supersaturated solutions of the model drug in the stomach, coupled to a two solid-state model to allow precipitation to the free form of the drug to moderate the extent of supersaturation obtained. The mechanistic salt model can be used to aid in screening and salt form selection to mitigate effects of ARAs. Further work is to include development of similar case studies for a variety of pharmaceutical salts, to adapt the model to handle pH-modifying excipients, and to add the salt and two-state mechanistic models to the SIVA Toolkit to facilitate biopharmaceutics IVIVE of salts.

## Figures and Tables

**Table 1:** Aqueous and Biorelevant solubility of the model drug [8]. For modelling purposes, it has been assumed that the quoted pH values are those at the end of the equilibration period and not the nominal pH values.

Medium	Solubility (mg/mL)	Medium	Solubility (mg/mL)
Aqueous		Aqueous	
pH 1.5	6.5	pH 8.5	0.021
pH 3.9	3.235	pH 9.1	0.03
pH 5.4	0.136	pH 10.6	0.442
pH 6.4	0.021	**Biorelevant**	
pH 6.6	0.019	FaSSIF (pH 6.5)	0.043
pH 7.5	0.015	FeSSIF V1 (pH 5)	0.99
pH 7.9	0.015		

**Table 2:** Input parameters for the model compound used in the PBPK simulations.

Parameters	Value	Reference/Comments
**Physicochemical Properties**		
Molecular weight (g/mol)	500	500–600 ^#^ Gesenberg et al. [8]
Compound Type	Ampholyte	Modelled as diprotic base
logP_o:w_	2.288	Back calculated from logD at pH 6.5 Gesenberg et al. [8]
pKa	6.25 (b), 2.1 (b), 9.8 (a)	Fitted in SIVA and based on Gesenberg et al. [8]
**Absorption Parameters**		
Formulation	Immediate Release	
Intrinsic solubility (mg/mL)	0.0142	Fitted in SIVA
Solubility factor	457.8	Fitted in SIVA
log*K_m:w,unionsed_*	4.34	Fitted in SIVA
log*K_m:w,ionised_*	3.97	Fitted in SIVA
Precipitation Model	Model 2	Simcyp default precipitation model
*CSR*	10	Simcyp default parameter
*PRC* (1/h)	4	Simcyp default parameter
Particle size distribution	Monodispersed	
Particle radius (microns)	1.958	Gesenberg et al. [8]
Particle dissolution model	Wang-Flanagan	
Particle handling model	Particle Population Balance (PPB)	
Particle *h_eff_* model	Hintz-Johnson	
Fluid Volumes Model	Simcyp advanced FVD (aFVD)	See main text
Gut Physiology	Simcyp “New Anatomy”	See main text
Permeability	Predicted	
Permeability model	MechPeff	
*P_trans,0_* (10^−6^ cm/s)	214.656	Predicted from logP_o:w_
*P_eff,man_* (10^−4^ cm/s) (Duodenum)	6.03 *	Predicted
*P_eff,man_* (10^−4^ cm/s) (Jejunum I)	8.27 *	Predicted
*P_eff,man_* (10^−4^ cm/s) (Jejunum II)	5.70 *	Predicted
*P_eff,man_* (10^−4^ cm/s) (Ileum I)	1.76 *	Predicted
*P_eff,man_* (10^−4^ cm/s) (Ileum II)	1.76 *	Predicted
*P_eff,man_* (10^−4^ cm/s) (Ileum III)	1.74 *	Predicted
*P_eff,man_* (10^−4^ cm/s) (Ileum IV)	1.67 *	Predicted
*P_eff,man_* (10^−4^ cm/s) (Colon)	0.58 *	Predicted
**Distribution and Elimination Parameters**		
*fu*	0.23	Simcyp Predicted
*B/P*	1.144	Simcyp predicted
Distribution Model	Minimal PBPK	
*Vss* (L/Kg)	0.78	Fitted to observed PO data (w/o famotidine)
Elimination *CLiv* (L/h)	10	Fitted to observed PO data (w/o famotidine)
Percentage *CL_H_* by 3A (%)	100	
*fu_gut_*	1	
**Trial Design**		
No of trials in each simulation	10	
No. of subjects in each trial	10	
Min. (Max.) age of subjects	20 (50)	
Proportion of females	0.5	
Simulation duration (h)	24	
Fluid volume taken with dose (mL)	250	Between subject CV on fluid volume intake was 0%
Dose (mg)	150	

^#^ For proprietary reasons Gesenberg et al. [8] did not provide the exact molecular weight of the model drug. Therefore, a sensitivity analysis was performed using a range of MWt from 500 to 600 g/mol. The simulated plasma concentration profiles were not sensitive within this range. * Population representative (i.e., average subject) regional *P_eff,man_*. Population variability is accounted for by the model during simulations. The values reported in the Table are without including the bile micelle-binding component; the effect of bile micelle binding on effective permeability is considered during simulations.

**Table 3:** Input parameters used in the PBPK model of the salt form of model compound.

Parameters	Value	Reference/Comments
**Solid State 1 (SS1) Parameters**		
Form	Salt	
Percentage in dose (%)	100	Refers to the % of SS1 in the dosage form prior to dosing.
Salt Solubility at *pH_max_* (mg/mL)	6.5	Free base highest solubility (pH 1.5)
*K_sp_* (mM^2^)	117.6	Back calculated from solubility at *pH_max_*
Intrinsic solubility, *S_o_* (mg/mL)	0.0142	Free base intrinsic solubility
Drug:Counterion Stoichiometry	1:1	
Concentration of counterion in drink (mg/mL)	0	
Counterion	Sulphate	
Counterion type	Strong acid	
Counterion *pKa1*	−3	
Counterion *pKa2*	1.92	
Particle surface solubility	Mechanistic Surface pH model	
Precipitation to SS1	Deselected	See main text for further explanation
**Solid State 2 (SS2) Parameters**		
Form	Free	
Percentage in dose (%)	0	
Intrinsic solubility, *S_o_* (mg/mL)	0.0142	Fitted in SIVA
Solubility factor (*SF*)	457.8	Fitted in SIVA
Precipitation to SS2	Permitted	
Precipitation Model	Model 2	
*CSR*	10	Default
*PRC* (1/h)	4	Default

**Table 4:** Simulated and Observed PK parameters (arithmetic means) of the model compound following administration of 150 mg of the free form under fasted condition without and with famotidine co-dosing.

Dosing Condition	C_max_ (mg/L)			AUC_0–t_ (mg/L∙h)			T_max_ (h)	
	Pred	Obs	Pred/Obs	Pred	Obs	Pred/Obs	Pred	Obs
Without Famotidine	1.22	1.23	0.99	9.80	8.15	1.20	1.3	3.0
With Famotidine	0.35	0.24	1.45	3.48	2.33	1.49	3.6	3.0

**Table 5:** Simulated PK parameters (arithmetic means ± SD) of model compound following administration of 150 mg of salt form under fasted condition without and with famotidine co-dosing.

Dosing Condition	C_max_ (mg/L)	AUC_0–t_ (mg/L·h)	T_max_ (h)
Without Famotidine	1.26 ± 0.51	9.8 ± 4.04	1.49 ± 0.58
With Famotidine	0.69 ± 0.31	6.19 ± 2.92	2.17 ± 1.15

## Data Availability

Not applicable.
